# Vascular endothelial growth factor inhibitor-induced cardiotoxicity: prospective multimodality assessment incorporating cardiovascular magnetic resonance imaging

**DOI:** 10.1136/heartjnl-2024-325535

**Published:** 2025-04-03

**Authors:** Stephen J H Dobbin, Kenneth Mangion, Colin Berry, Giles Roditi, Susmita Basak, John D McClure, Katriona Brooksbank, Piotr Sonecki, Steven Sourbron, Jeff Evans, Jeff White, Paul Welsh, Elaine Butler, Balaji Venugopal, Rhian M Touyz, Robert J Jones, Mark C Petrie, Ninian N Lang

**Affiliations:** 1 University of Glasgow BHF Glasgow Cardiovascular Research Centre, Glasgow, UK; 2 Cardiology, Golden Jubilee National Hospital, Clydebank, UK; 3 Department of Radiology, Glasgow Royal Infirmary, Glasgow, UK; 4 UK Research and Innovation, Didcot, UK; 5 Queen Elizabeth University Hospital, Glasgow, UK; 6 Department of Infection, Immunity and Cardiovascular Disease, The University of Sheffield—Western Bank Campus, Sheffield, UK; 7 School of Cancer Sciences, University of Glasgow, Glasgow, UK; 8 Research Institute of McGill University Health Centre, McGill University, Montreal, Quebec, Canada

**Keywords:** Hypertension, Magnetic Resonance Imaging, Heart Failure

## Abstract

**Background:**

Vascular endothelial growth factor inhibitors (VEGFIs) are effective anticancer agents, but are associated with cancer therapy-related cardiac dysfunction (CTRCD) and hypertension. The timing, frequency and magnitude of these toxicities are poorly defined. The objective of this study is therefore to investigate the incidence, time course and mechanisms of VEGFI-associated CTRCD and hypertension.

**Methods:**

Patients commencing VEGFI underwent blood pressure (BP) monitoring, echocardiography and cardiac biomarker measurement at baseline and prospectively over 24 weeks. Serial adenosine stress perfusion cardiovascular MRI (CMR) was performed in a substudy. CTRCD was defined as left ventricular ejection fraction (LVEF) decline by ≥10 percentage points from baseline to a value <50%.

**Results:**

78 patients participated (68% men; age 63±11 years). 15 patients (19%) developed CTRCD, and it was evident at 4 weeks in 93% of cases. Overall, LVEF was 4.2% (95% CI: −6.2% to −2.3%, p<0.001) lower than baseline at 4 weeks. At 4 weeks, N-terminal pro-brain natriuretic peptide, but not troponin, was higher in patients with CTRCD. 62 (77%) patients developed hypertension. Home systolic and diastolic BP increased by 7.2 mm Hg (4.7–9.8, p<0.001) and 4.8 mm Hg (3.1–6.5, p<0.001), respectively, at 1 week. There was no association between change in LVEF and BP.

CMR-derived LVEF, T1 relaxation times and resting myocardial blood flow (n=46) were 5.2% (−7.3% to −3.1%, p<0.001), 27 ms (−40 to −14, p<0.001) and 14.7 mL/100mL/min (−24.2 to −5.1, p=0.004), respectively, lower at 4 weeks.

**Conclusion:**

VEGFI-associated CTRCD is frequent and occurs early. This finding has implications for prioritising early cardiac imaging follow-up after commencing treatment. Underlying mechanisms include myocardial and microvascular effects that are at least partly independent of hypertension.

**WHAT IS ALREADY KNOWN ON THIS TOPIC**

- Vascular endothelial growth factor inhibitors are effective anticancer agents, but they are associated with a range of cardiovascular toxicities, including hypertension and left ventricular dysfunction.

**WHAT THIS STUDY ADDS**

- This prospective study defines the timing and incidence of vascular endothelial growth factor inhibitors (VEGFIs)-associated hypertension and cardiac dysfunction.
- This study is the first to examine changes in left ventricular function with VEGFIs by cardiac MRI and provides insights into the underlying mechanisms, particularly the potential role of microvascular dysfunction.

**HOW THIS STUDY MIGHT AFFECT RESEARCH, PRACTICE OR POLICY**

- This study provides data to assist in the development of monitoring strategies as well as therapeutic strategies for prevention and management of VEGFI-associated cardiac dysfunction.

## Introduction

 Vascular endothelial growth factor inhibitors (VEGFIs) are used to treat a wide range of cancers. They exploit the dependency of tumours on their blood supply by inhibiting angiogenesis, the process of new blood vessel formation. However, these oncological benefits are accompanied by unwanted cardiovascular toxicities including hypertension, left ventricular systolic dysfunction (LVSD) and heart failure (HF). The true incidence, timing and consequences of these adverse effects are not well-described. Clinical trial assessment and reporting of the cardiovascular effects of these agents are heterogeneous, and trial participants may not be representative of the wider population of patients ultimately treated with them.^1^ Retrospective analysis and registry data^2^ provide some understanding, but prospective studies examining the cardiovascular effects of these drugs have been limited.

Other than our own previous small pilot study,^3^ no prospective study has used serial cardiac MRI, which has the potential to gain more accurate insight into the mechanisms of the cardiac effects of VEGFI.

In this prospective study, our objectives were to investigate the incidence, time course, risk factors and mechanisms of VEGFI cancer therapy-related cardiac dysfunction (CTRCD) and hypertension. In addition to detailed blood pressure (BP) assessment, the incidence and characteristics of VEGFI-associated CTRCD were investigated using cardiac biomarkers and multimodality imaging techniques including echocardiography and stress perfusion cardiac MRI (CMR).

## Methods

This was a prospective observational study of patients with cancer attending a regional cancer network (West of Scotland Cancer Network). Patients with renal cell cancer (RCC), hepatocellular carcinoma (HCC), sarcoma and thyroid cancer being considered for VEGFI therapy, alone or in combination with immune checkpoint inhibitors, were screened for participation. Patients were recruited between April 2019 and April 2021.

**Figure 1:**
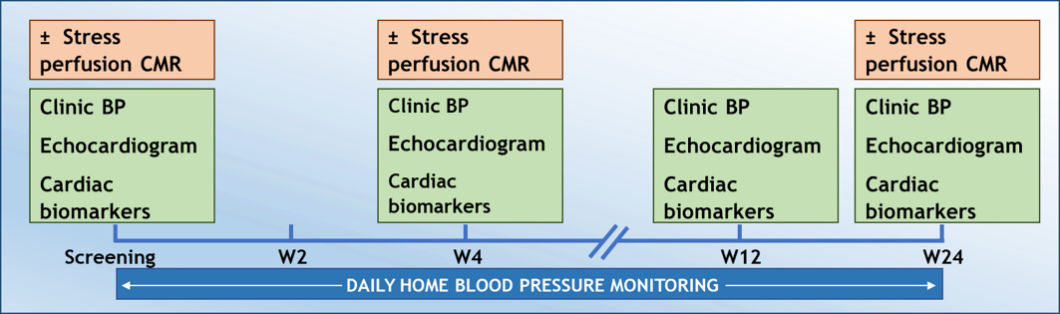
Study schedule. BP, blood pressure; CMR, cardiac MRI.

Baseline assessments were made before starting VEGFI therapy and repeated at 4, 12 and 24 weeks (±2 weeks) after starting treatment. In the CMR substudy, MRI scans were performed at baseline, 4 and 24 weeks (±2 weeks) (figure 1). Decisions around VEGFI dose initiation, modification, interruption or discontinuation as well as the initiation of antihypertensive or other cardiovascular medications were at the discretion of the clinical oncology team.

### Echocardiography

Transthoracic echocardiography was performed using a Siemens Acuson SC2000 scanner (Siemens Healthcare, Erlangen, Germany) and acquired and reported according to a standardised British Society of Echocardiography guideline-based protocol.^4^ Left ventricular ejection fraction (LVEF) was assessed by Simpson’s biplane method and by global longitudinal strain (GLS) using two-dimensional speckle tracking via the vector velocity imaging method. Interobserver and intraobserver variability were measured by comparing measurements of a random sample of 10% of scans.

### BP monitoring

BP monitoring was conducted via ‘clinic’ and home BP measurements (Omron M2, Omron Healthcare, the Netherlands). Home measurements were taken once a day, and a rolling 7-day average BP measurements was calculated.

### Stress perfusion CMR

Adenosine stress perfusion CMR scans were performed on a 3.0 Tesla MRI scanner (MAGNETOM Prisma, Siemens Healthcare, Germany) using a standardised stress perfusion protocol (online supplemental figure 1). Hyperaemia was confirmed by a haemodynamic response defined as systolic blood pressure (SBP) decrease>10 mm Hg and heart rate increase>10 beats per minute and/or the onset of typical symptoms (dyspnoea, chest tightness, flushing).

CMR analysis was performed with commercially available software (cvi42, V.5.9.4, Circle Cardiovascular Imaging, Canada). MRI data were analysed blinded to patient identifiable data as well as the date and time of the scans. Quantitative analysis of first-pass perfusion was analysed with open-source software PMI V.0.4 (Platform for Research Imaging, Leeds, UK).

### Biomarker analysis

High sensitivity troponin T (hs-TnT) and N-terminal pro-brain natriuretic peptide (NT-proBNP) were analysed using Elecsys Troponin T hs and Elecsys proBNP II assays (both F. Hoffmann-La Roche, Switzerland), on a Cobas e411 analyser. hs-TnT>99th centile for the assay (>14 pg/mL) and NT-proBNP>125 pg/mL were considered abnormal, in keeping with European Society of Cardiology (ESC) Cardio-Oncology guidelines.^5^

### Definitions for VEGFI-induced cardiovascular toxicities

VEGFI-induced hypertension was defined as a rolling 7-day average home BP of ≥135/≥85 mm Hg, where at least 3 days of measurements were recorded in a 7-day period, in line with international guidelines and/or the initiation or escalation of existing antihypertensive therapy.^6^,^8^ In patients whose BP was above this threshold at baseline, VEGFI-induced hypertension was defined as an increase in SBP or diastolic blood pressure (DBP) of ≥20 mm Hg from baseline^7 9^ and/or the initiation or escalation of antihypertensive therapy.

CTRCD was defined as per International Cardio-Oncology Society definitions and ESC guidelines. Asymptomatic ‘moderate’ CTRCD was defined as a reduction in LVEF of ≥10% to a value <50% and ≥40% or a reduction in LVEF of <10% to a value <50% and ≥40% in conjunction with a >15% reduction in GLS and/or a rise in cardiac biomarkers (hs-TnT or NT-proBNP). Asymptomatic ‘severe’ CTRCD was defined as a drop in LVEF to <40%.^5 10^ As the clinical significance of ‘mild’ asymptomatic CTRCD is uncertain, this was not included in the principal definition of VEGFI-induced CTRCD.

The Heart Failure Association–International Cardio-Oncology Society (HFA-ICOS) risk assessment tool was used to determine baseline risk of VEGFI-induced CTRCD.^5 11^

### Statistical analysis

Categorical variables are expressed as number and percentage. Continuous variables with normal distribution are expressed as mean±SD, while those without normal distribution are expressed as median±IQR. Differences between independent groups were assessed using t-tests, Mann-Whitney tests, Wilcoxon signed-rank test or Fisher’s tests where appropriate. Repeated measures mixed-effect models were used to examine the change in outcomes over time according to CTRCD and VEGFI-induced hypertension status. Results are presented as the least square mean with 95% CI at each timepoint. Models were adjusted for baseline LVEF, visit, CTRCD status and interaction of CTRCD status and visit with a random intercept and slope per patient with an unstructured covariance structure. Linear regression models adjusted for baseline LVEF were used to examine associations between individual baseline factors or biomarkers and changes in LVEF. Limited pre-existing data on the incidence of VEGF-associated CTRCD meant that a formal power calculation was not appropriate. A p-value<0.05 was taken to indicate a statistically significant effect. All analyses were performed using Stata V.16.0 or higher (Stata Corp, College Station, Texas, USA).

### Patient and public involvement

The study was developed in consultation with patients with cancer and clinicians. The study was presented to the West of Scotland Cancer Centre Clinical Trials Executive Committee, which includes patient representatives, and feedback was acted upon prior to final approval.

## Results

### Study patients

78 patients underwent follow-up assessments (online supplemental figure 2). The mean age was 62.7±11.3 years. The majority of patients were men (68%) and cardiovascular comorbidities were prevalent at baseline, including 41% with a history of hypertension. Mean baseline home SBP was 128±14 mm Hg and home DBP was 78±10 mm Hg, while mean LVEF was 63.1%±7.7%. 45% would be considered ‘high’ or ‘very high’ risk according to the HFA-ICOS baseline cardiotoxicity risk calculator, and 49% were treated with VEGFI combined with immunotherapy (table 1).

**Table 1:** Baseline characteristics in patients with and without VEGFI-induced CTRCD

	All patients n=78	CTRCD n=15	No CTRCD n=63	P value
Age, years; mean±SD	63±11	64±11	62±12	0.58
Sex, n (%) Female	25 (32)	8 (53)	17 (27)	0.049
BMI, kg/m^2^; median (IQR)	27 (25–31)	28 (24–30)	27 (25–31)	0.83
Cancer primary site, n (%)				0.64
Renal cell	56 (72	10 (67)	46 (73)	
Hepatocellular	13 (17)	2 (13)	11 (18)	
Sarcoma	5 (6)	2 (13)	3 (5)	
Thyroid	4 (5)	1 (7)	3 (5)	
Metastases, n (%)	67 (86)	13 (87)	54 (86)	0.92
Previous cancer treatment, n (%)				
Surgery	49 (63)	10 (67)	39 (62)	0.73
Radiotherapy	13 (17)	3 (20)	10 (16)	0.7
Chemotherapy	12 (15)	4 (27)	8 (13)	0.18
HR, beats per minute; mean±SD	77±15	76±12	78±16	0.74
Home SBP, mm Hg; mean±SD	128±14	125±13	129±14	0.4
Home DBP, mm Hg; mean±SD	78±10	71±9	79±10	0.013
LVEF, %; mean±SD	63±8	59±7	64±8	0.015
LVIDd, mm; mean±SD	46±5	46±5	46±5	0.94
hs-TnT, pg/mL; median (IQR)	9.7 (6.1–15.4)	8.8 (6.5–19.7)	9.8 (6.0–15.3)	0.58
NT-proBNP, pg/mL; median (IQR)	132 (57–289)	375 (76–984)	106 (57–222)	0.037
HFA-ICOS risk assessment, n (%)				0.089
Low	19 (24)	1 (7)	18 (29)	
Medium	24 (31)	3 (20)	21 (33)	
High	17 (22)	5 (33)	12 (19)	
Very high	18 (23)	6 (40)	12 (19)	
Medical history, n (%)				
Heart failure	1 (1)	0 (0)	1 (2)	0.62
Hypertension	32 (41)	4 (27)	28 (44)	0.21
Coronary artery disease	11 (14)	5 (33)	6 (10)	0.017
Myocardial infarction	9 (12)	3 (20)	6 (10)	0.25
PCI or CABG	7 (9)	2 (13)	5 (8)	0.51
Diabetes	10 (13)	2 (13)	8 (13)	0.95
Atrial fibrillation	4 (5)	1 (7)	3 (5)	0.76
Stroke	5 (6)	1 (7)	4 (6)	0.96
Dyslipidaemia	9 (12)	3 (20)	6 (10)	0.25
Medications, n (%)				
VEGFI				0.44
Axitinib	37 (47)	6 (40)	31 (49)	
Lenvatinib	14 (18)	2 (13)	12 (19)	
Pazopanib	13 (17)	3 (20)	10 (16)	
Sunitinib	6 (8)	2 (13)	4 (6)	
Sorafenib	3 (4)	0 (0)	3 (5)	
Tivozinib	3 (4)	1 (7)	2 (3)	
Cabozantinib	1 (1)	0 (0)	1 (2)	
Bevacizumab	1 (1)	1 (7)	0 (0)	
VEGFI group				0.86
VEGFI monotherapy	40 (51)	8 (53)	32 (51)	
Combined VEGFI immunotherapy	38 (49)	7 (47)	31 (49)	
Beta-blocker	16 (21)	5 (33)	11 (18)	0.17
ACEi/ARB	19 (24)	2 (13)	17 (27)	0.27
Dihydropyridine CCB	18 (23)	4 (27)	14 (22)	0.71
Nitrate	1 (1)	0 (0)	1 (2)	0.62
Loop diuretic	5 (6)	1 (7)	4 (6)	0.96
Thiazide diuretic	4 (5)	1 (7)	3 (5)	0.76
MRA	1 (1)	0 (0)	1 (2)	0.62

ACEi, ACE inhibitor; ARB, angiotensin receptor blocker; BMI, body mass index; CABG, coronary artery bypass grafting; CCB, calcium channel blocker; CTRCD, cancer therapy-related cardiac dysfunction; DBP, diastolic blood pressure; HFA-ICOS, Heart Failure Association–International Cardio-Oncology Society; HR, heart failure; hs-TnT, high sensitivity troponin T; LVEF, left ventricular ejection fraction; LVIDd, diastolic left ventricular internal diameter; MRA, mineralocorticoid receptor antagonist; NT-proBNP, N-terminal pro-brain natriuretic peptide; PCI, percutaneous coronary intervention; SBP, systolic blood pressure; VEGFI, vascular endothelial growth factor inhibitor.

55 patients (71%) completed the full protocol-specified follow-up of 24 weeks (online supplemental figure 2). 49 of these patients (89%) were still receiving VEGFI therapy at 24 weeks.

### LV function by echocardiography

**Figure 2:**
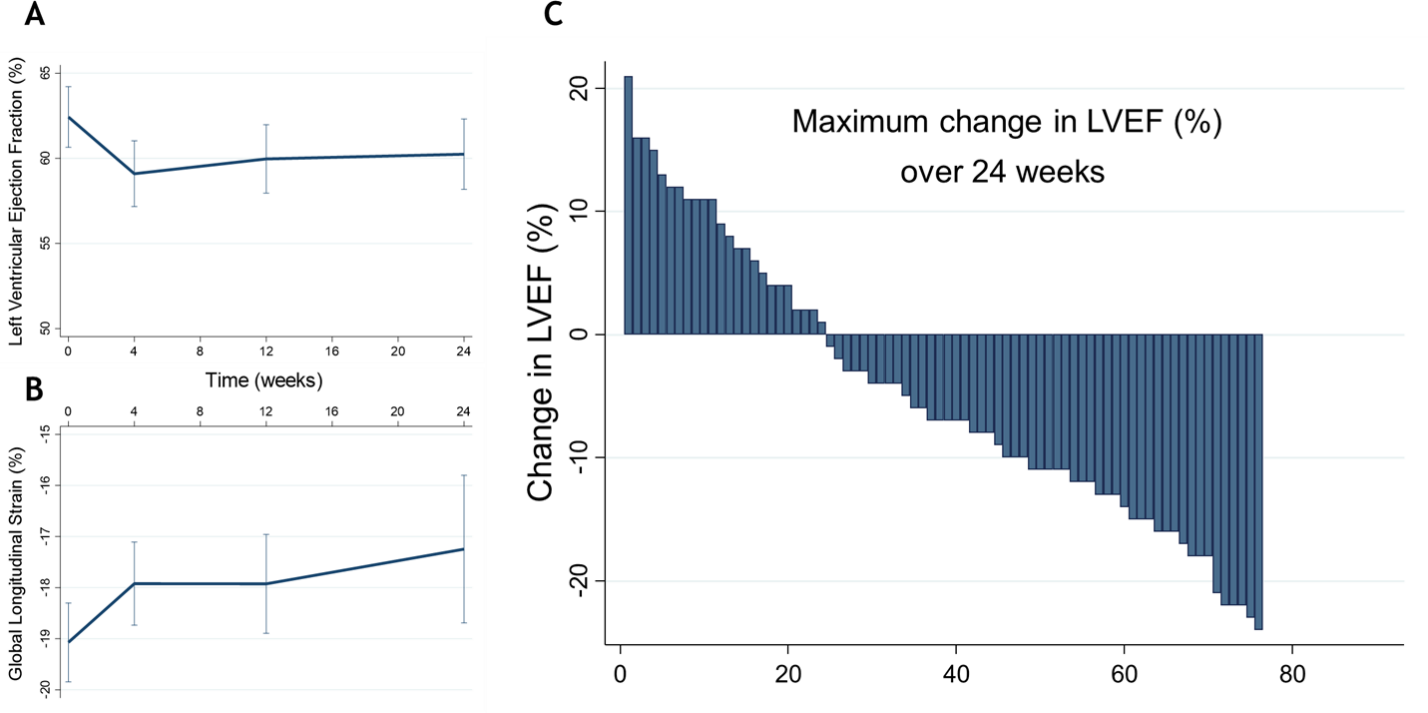
LVEF (A), global longitudinal strain (B) and maximal change in left ventricular ejection fraction (C) in all patients treated with VEGFI therapy. LVEF, left ventricular ejection fraction.

By 4 weeks, LVEF was 4.2% (95% CI: −6.2% to −2.3%, p<0.001) lower than baseline. At 12 weeks, LVEF remained lower than baseline (figure 2A). LV GLS was impaired in comparison to baseline at each timepoint (online supplemental table 1 and figure 2B).

### Incidence and clinical course of CTRCD

15 patients (19%) developed at least moderate CTRCD. 11 met criteria based on a reduction in LVEF of ≥10% to a value <50% from baseline. Two patients met criteria for ‘severe’ CTRCD with a drop in LVEF to <40%. Five patients developed symptomatic HF. Of the patients who met criteria for at least moderate CTRCD, 93% did so at 4 weeks when compared with baseline. In those patients, LVEF was 11.3% (95% CI: −15.9% to −6.6%), p<0.001 lower at 4 weeks in comparison to baseline. The greatest change in LVEF in each patient treated with VEGFI is displayed in figure 2C.

After meeting the criteria for CTRCD, LVEF subsequently improved by ≥10 percentage points in eight patients (53%), five of whom had recovery of LVEF to baseline values. The management of VEGFi-associated CTRCD was heterogeneous and frequently directed by oncologic and comorbidity considerations, but the majority received treatment with neurohormonal antagonists. Seven patients had a temporary interruption of VEGFI treatment with subsequent resumption of VEGFI at a lower dose, while three patients had VEGFI discontinued altogether. Patients with CTRCD were more likely to be women and have a history of coronary artery disease with lower baseline DBP and LVEF (table 1).

### Blood pressure

**Figure 3:**
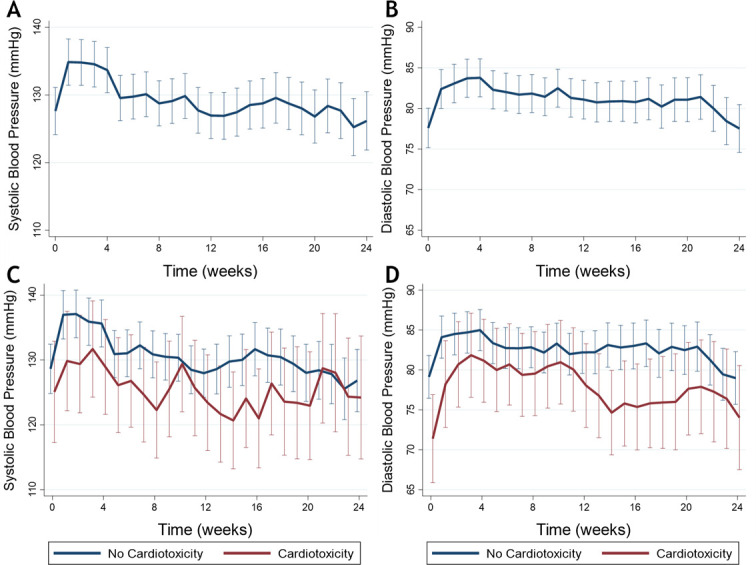
Home systolic and diastolic blood pressure in all patients treated with vascular endothelial growth factor inhibitors (VEGFI) therapy (A,B) and in those with and without VEGFI-induced cancer therapy-related cardiac dysfunction (C,D).

Home SBP and DBP were 7.2 mm Hg (95% CI: 4.7 to 9.8, p<0.001) and 4.8 mm Hg (95% CI: 3.1 to 6.5, p<0.001), respectively, higher at 1 week after starting VEGFI, and these changes persisted at 4 weeks but SBP and DBP were not different to baseline thereafter (online supplemental table 1 and figure 3A,B). 62 patients (79%) met criteria for VEGFI-induced hypertension during follow-up. The management of VEGFI-induced hypertension was directed by the oncology team using a non-dihydropyridine calcium channel blocker, an ACE inhibitor or a combination of these.

In contrast to home BP measurement, clinic SBP measurements did not change significantly following the initiation of VEGFI therapy when compared with baseline. Although clinic DBP was 3.3 mm Hg (95% CI: 1.1 to 5.6, p=0.004) higher at 4 weeks compared with baseline, this was not maintained at 12 and 24 weeks.

### CTRCD and hypertension

Of the 15 patients who met criteria for CTRCD, 10 (67%) also met criteria for VEGFI-induced hypertension. Home BP was not different in those with CTRCD in comparison to those without (figure 3C,D). There was also no difference in LVEF or LV GLS changes over time in those who developed hypertension and those who did not (online supplemental figure 3).

### Cardiac biomarkers

**Figure 4:**
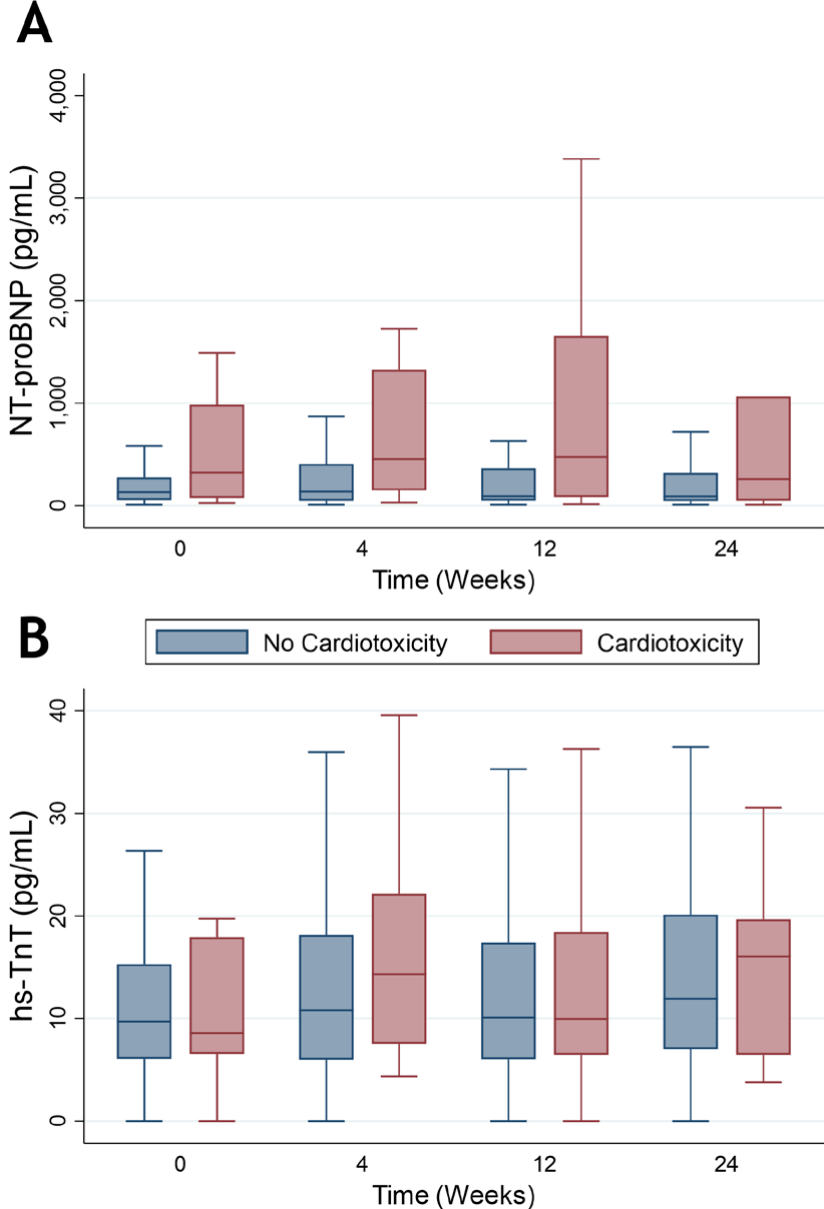
N-terminal pro-brain natriuretic peptide (NT-proBNP) (A) and high-sensitivity troponin T (hs-TnT) (B) in those with and without vascular endothelial growth factor inhibitor-induced cancer therapy-related cardiac dysfunction.

Overall, NT-proBNP was 318 pg/mL (95% CI: 51 to 584, p=0.02) higher at 4 weeks than it was at baseline. At 4 weeks, NT-proBNP was 1318 pg/mL (95% CI: 629 to 2007, p<0.001) higher in those who met criteria for CTRCD than baseline, but this was not maintained at 12 and 24 weeks (figure 4A). There were no changes in NT-proBNP in those who did not meet criteria for CTRCD (online supplemental table 1). There was no significant change in hs-TnT at any timepoint with no difference between those with and without CTRCD (figure 4B).

### Cancer and treatment groups

There were no differences in any BP, imaging or biomarker measures between patients with RCC and those with other cancers (HCC, thyroid and sarcoma) (online supplemental figure 4), nor between patients treated with VEGFI monotherapy and those treated with combined VEGFI and immunotherapy (online supplemental figure 5).

### CMR substudy

**Figure 5:**
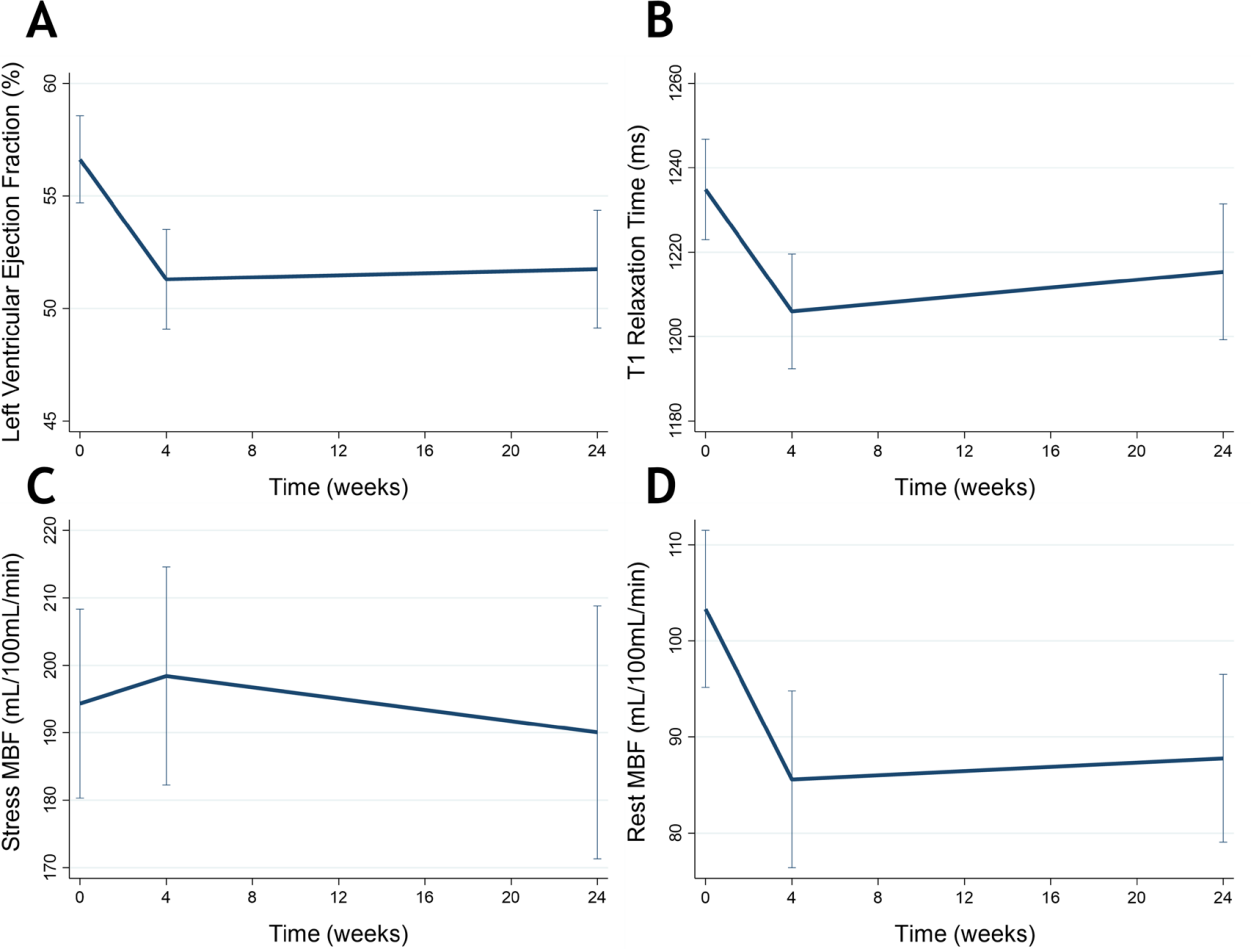
Left ventricular ejection fraction (A), T1 relaxation times (B) and resting (C) and adenosine stress (D) myocardial perfusion with vascular endothelial growth factor inhibitor therapy as measured by cardiovascular MRI. MBF, myocardial blood flow.

46 patients (59% of the total cohort) underwent serial stress perfusion CMR. Baseline demographics for this group were similar to those of the overall study population (online supplemental table 2). Overall, there was a reduction of 5.2% (95% CI: −7.3% to −3.1%, p<0.001) in CMR LVEF at 4 weeks from baseline with VEGFI therapy, which persisted at 24 weeks (figure 5A).

### Myocardial tissue characteristics

Native T1 relaxation times were 27 ms (95% CI: −40 to −14, p<0.001) lower at 4 weeks than baseline (figure 5B). T1 times in patients with CTRCD fell by 32 ms (95% CI: −58 to −6, p=0.017) at 4 weeks and 40 ms (95% CI: −70 to −9, p=0.013) at 24 weeks compared with baseline (online supplemental figure 6B) while there were no changes in those without CTRCD (online supplemental table 1).

### Myocardial perfusion

Resting myocardial blood flow (MBF) was 14.7 mL/100 mL/min (95% CI: −24.2 to −5.1, p=0.004) lower at 4 weeks when compared with baseline (figure 5C). In patients with CTRCD, the reduction in resting MBF remained lower than baseline over 24 weeks (online supplemental figure 6C). In those without CTRCD, resting MBF was lower at 4 weeks, but there was no evidence of a difference at 24 weeks compared with baseline. There were no changes in stress MBF at 4 or 24 weeks compared with baseline (figure 5D and online supplemental figure 6D).

## Discussion

This prospective study was designed to investigate the cardiovascular effects of VEGFI in routine oncology treatment for a variety of cancer types. The use of multimodality imaging techniques including stress perfusion CMR provide novel insights into the incidence and myocardial effects of VEGFI. In this clinically relevant cohort, there was a high incidence of VEGFI-induced hypertension, but our findings suggest that VEGFI-associated CTRCD may not be explained by hypertension alone. The vast majority of cases of CTRCD were first evident at 4 weeks and appeared to be at least partially reversible. NT-proBNP was higher in patients with CTRCD and may be a useful biomarker of baseline and incident risk for VEGFI-induced CTRCD.

At least moderate VEGFI-induced CTRCD occurred in 19% of patients. This is higher than has previously been reported and may reflect the comprehensive imaging follow-up protocol, our use of robust CTRCD definitions and the prospective assessment of ‘real world’ patients with baseline comorbidity, with a range of primary cancer sites treated with a variety of VEGFI drugs. CTRCD resulting from VEGFI therapy may have been under-reported in clinical trials.^1^ The incidence of asymptomatic LVSD with VEGFI therapy has been reported to be as low as 2.4% in a meta-analysis of 10 647 patients in clinical trials. However, of the 21 trials included, only 5 had baseline and protocol-mandated follow-up cardiac imaging.^12^ Additionally, in a previous echocardiography-based study of 90 patients with metastatic RCC treated with the VEGFI sunitinib, the incidence of CTRCD was 9.7%.^2 13^

Importantly, almost all patients who developed CTRCD did so by 4 weeks and following interventions with neurohormonal antagonists and/or VEGFI dose modification, there was improvement in LV function in approximately half of those affected. Given the frequency, timing and potential reversibility of VEGFI-associated CTRCD, these findings suggest that after pre-VEGFI assessments, priority should be given to early assessment of left ventricular function (ca. 4 weeks) after starting VEGFI. This strategy could allow for optimal detection of VEGFI-associated CTRCD and, importantly, may offer the opportunity to make meaningful interventions to improve LV function before potentially irreversible effects. This could simultaneously increase the chance of longer-term optimal cancer therapy delivery unimpeded by CTRCD, as well as reducing the risk of consequent HF. Our findings suggest that if LV function remains stable 4 weeks after starting VEGFI treatment, routine cardiac imaging may be of less value thereafter. This is relevant to future iterations of oncology and cardio-oncology guidelines.

A deterioration in LV GLS is frequently used as a marker of early detection of CTRCD in patients receiving a wide variety of anticancer drugs.^14 15^ To the best of our knowledge, GLS has not previously been assessed prospectively in patients receiving VEGFI. While moderate or severe CTRCD and a decline in GLS occurred within 4 weeks of starting VEGFI therapy, it is unclear if deterioration of LV GLS may occur even earlier or if it could be predictive of CTRCD. However, given that GLS can be affected substantially by cardiac loading conditions, it may have lower utility in the assessment of patients treated with VEGFI given the high incidence of VEGFI-associated hypertension.

Other than our own small pilot study,^3^ this is the first to use comprehensive serial CMR to assess longitudinal changes in cardiac function as well as myocardial tissue characteristics and perfusion. VEGFI treatment was associated with lower myocardial T1 relaxation times and myocardial perfusion, and these changes were more pronounced in patients with CTRCD. Typically, myocardial fibrosis and LV dysfunction are associated with prolonged T1 relaxation times,^16 17^ but preclinical studies suggest that VEGFI therapy is associated with mitochondrial dysfunction rather than apoptosis.^18^,^20^ In mice treated with sunitinib, cardiomyocytes exhibited mitochondrial swelling with degenerative changes including whorls and loss of cristae.^18^ Similarly, endomyocardial biopsies from patients with VEGFI-induced CTRCD have been reported to show mitochondrial dysfunction and not apoptosis or fibrosis with improvement in mitochondrial and cardiac function with HF therapies.^18 20^ T1 relaxation times reflect intracapillary plasma volume as well as extracellular matrix water content,^21^ and we hypothesise that the rapid reduction in T1 times observed here may represent a reduction in intracapillary volume. This is supported by our other observation that resting MBF is reduced following VEGFI exposure. Rarefaction, the loss of capillaries, has been observed in nail-fold capillaries of VEGFI-treated patients.^22 23^ Our study provides the first evidence to suggest that a similar process may occur within the coronary microvasculature and contribute to the development of VEGFI-associated CTRCD. While our study did not make a direct assessment of this, it is possible that VEGFI-induced effects on the sympathetic nervous system could contribute both to changes seen in measures of MBF and systemic BP effects. There were no changes observed in adenosine-induced stress MBF when compared with baseline, which suggests that vasodilatation may reverse the microvascular changes associated with VEGFI therapy and that, at least for the duration of follow-up, this is a functional (vasoconstrictor) response rather than complete capillary loss. This may have potential therapeutic implications for the treatment and prevention of VEGFI-induced CTRCD.

There was a high incidence of VEGFI-associated hypertension, with 83% of patients meeting criteria during follow-up. While VEGFI-associated hypertension has been reported in up to 80% of patients previously,^24^ the majority of prior studies have reported an incidence of 30–60%.^25 26^ We demonstrated an early rise in BP at 1 week which has important implications for BP surveillance, including the use of HBPM in all patients starting VEGFI treatment.^27^

It has previously been proposed that VEGFI-induced CTRCD may be a direct consequence of the rise in BP and increased cardiac afterload.^28 29^ However, we did not demonstrate any correlation between SBP and changes in LVEF in this study (online supplemental figure 3). Therefore, although the development of de novo or worsening hypertension may contribute to the development of CTRCD, these findings suggest that major pathophysiological mechanisms of VEGFI-induced CTRCD include effects that are independent of BP and afterload.

There are some limitations to this study. Not all patients underwent both imaging modalities, which may limit the generalisability of the findings. However, given the comprehensive use of echocardiography in clinical practice, we focused on this aspect of imaging. Additionally, the substantial use of CMR in this study provides the gold standard of LV functional assessment, as well as further mechanistic insights. Eligibility criteria were intentionally broad to allow representation of ‘real world’ patients receiving VEGFI, but this does introduce some baseline heterogeneity and limits subgroup assessment. Due to the nature of this population of patients with potentially advanced malignancy, there are some missing data and follow-up, although this reflects the ‘real world’ and was lower than has previously been reported.^13^ This missing data may be open to bias. While a sensitivity analysis could allow this to be investigated, it would require a much larger sample size to be an effective approach. We appreciate that although our prospective assessments were comprehensive, some are limited by this sample size. Additionally, the study follow-up precludes any conclusions on long-term effects of VEGFI and further study is required to examine the longer-term cardiovascular impact of these agents.

## Conclusions

In this detailed prospective study of patients treated with a range of VEGFI, we have demonstrated that VEGFI-induced CTRCD is very common. When it occurs, this is generally early during treatment and not clearly attributable to coexisting prohypertensive effects. VEGFI-induced changes in myocardial perfusion may represent a pathophysiological mechanism and potential therapeutic target for prevention and treatment of VEGFI-induced CTRCD. Our findings support the use of home BP monitoring and the adoption and prioritisation of very early cardiac imaging follow-up for patients treated with these drugs.

## Supplementary material

10.1136/heartjnl-2024-325535 online supplemental file 1

## Data Availability

Data are available on reasonable request.
