# Supplementary material for: Vascular endothelial growth factor inhibitor-induced cardiotoxicity: prospective multimodality assessment incorporating cardiovascular magnetic resonance imaging
Source: Heart. 2025 Apr 3;111(19):e325535. doi: 10.1136/heartjnl-2024-325535 (PMC12505041; doi:10.1136/heartjnl-2024-325535)
Supplement: online supplemental file 1 [file heartjnl-111-19-s001.docx]

**Supplementary Data**

**Figure legends**

**Supplementary Figure 1.** CMR protocol. bSSFP – balanced steady-state free precession, HLA – horizontal long axis, LVOT – left ventricular outflow tract, MOLLI – modified Look-Locker inversion-recovery, SA – short axis, VLA – vertical long axis.

**Supplementary Figure 2.** Consort diagram of study recruitment. CMR – cardiac magnetic resonance

**Supplementary Figure 3.** Left ventricular ejection fraction (A) and global longitudinal strain (B) in patients who developed VEGFI-associated hypertension and those who did not.

**Supplementary Figure 4.** Systolic (A) and diastolic (B) blood pressure, left ventricular ejection fraction (C) and global longitudinal strain (D) in patients with RCC and other cancers treated with VEGFI therapy.

**Supplementary Figure 5.** Home systolic (A) and diastolic (B) blood pressure, left ventricular ejection fraction by echocardiography (C) and by CMR (D), T1 relaxation times (E), resting (F) and adenosine stress (G) myocardial perfusion and resting (H) and adenosine stress (I) vascular permeability in patients treated with VEGFI monotherapy and combined VEGFI with immunotherapy.

**Supplementary Figure 6.** Left ventricular ejection fraction by CMR (A), T1 relaxation times (B), resting (C) and adenosine stress (D) myocardial perfusion and resting (E) and adenosine stress (F) vascular permeability in patients who developed VEGFI-associated CTRCD and those who did not.

**Supplementary Figure 1.** CMR protocol. bSSFP – balanced steady-state free precession, HLA – horizontal long axis, LVOT – left ventricular outflow tract, MOLLI – modified Look-Locker inversion-recovery, SA – short axis, VLA – vertical long axis.

**
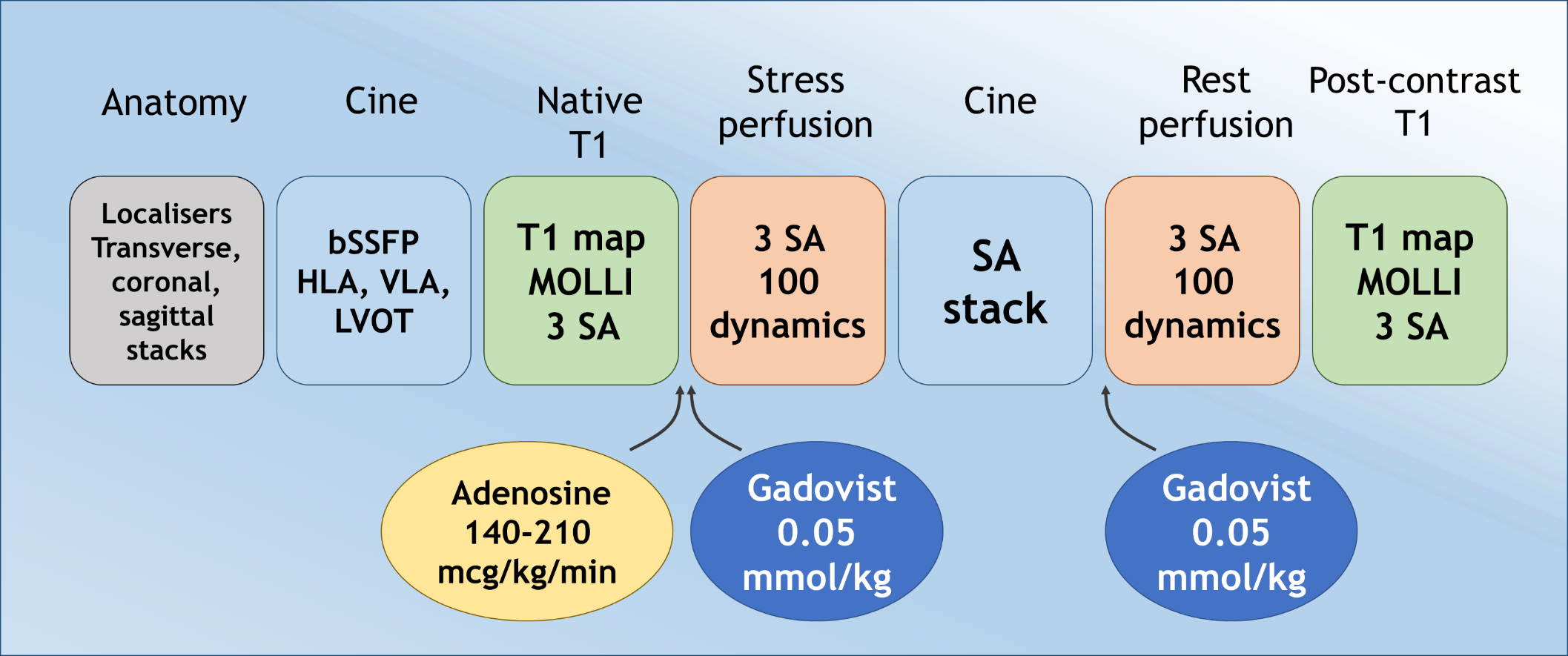
**

**Supplementary Figure 2.** Consort diagram of study recruitment.

CMR – cardiovascular magnetic resonance

**
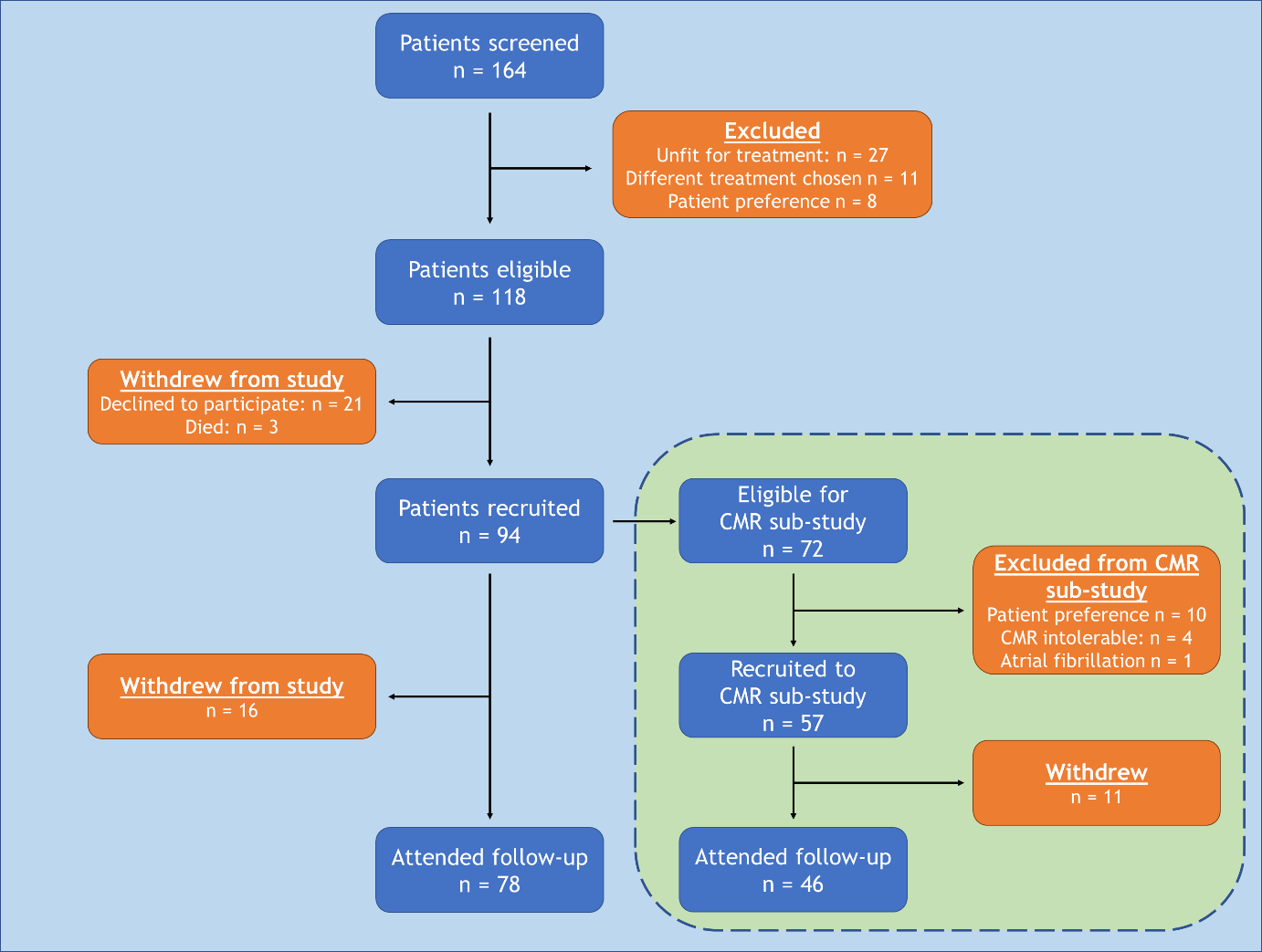
**

**Supplementary Figure 3.** Left ventricular ejection fraction (A) and global longitudinal strain (B) in patients who developed VEGFI-associated hypertension and those who did not.


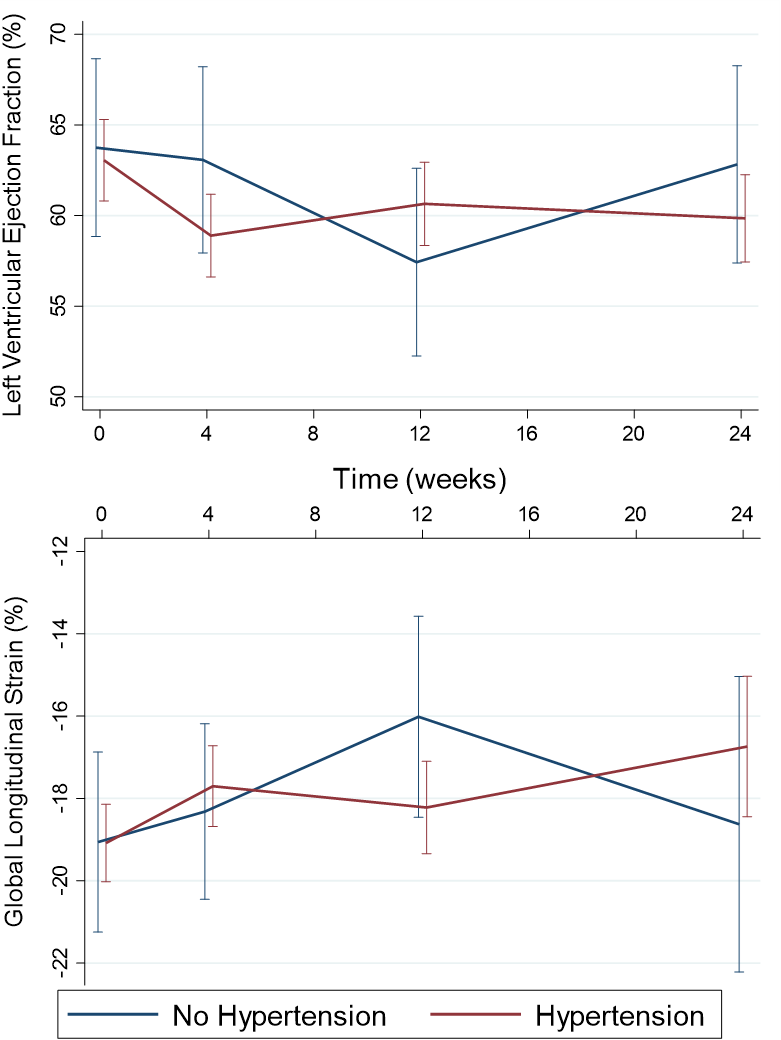


**Supplementary Figure 4.** Home systolic (A) and diastolic (B) blood pressure, left ventricular ejection fraction (C) and global longitudinal strain (D) in patients with RCC versus other cancers treated with VEGFI therapy.

**
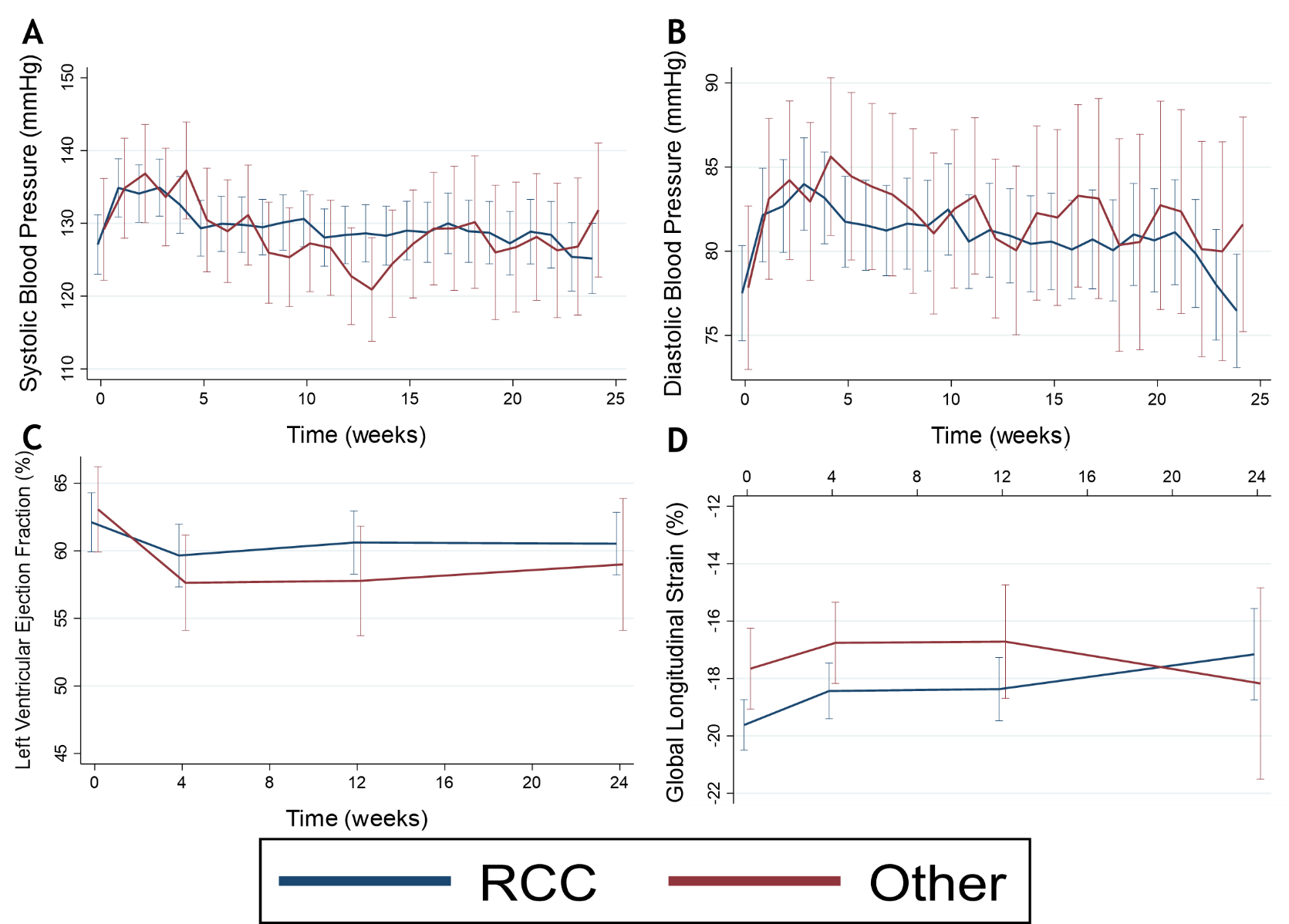
**

**Supplementary Figure 5.** Home systolic (A) and diastolic (B) blood pressure, left ventricular ejection fraction by echocardiograph (C) and by CMR (D), T1 relaxation times (E), resting (F) and adenosine stress (G) myocardial perfusion and resting (H) and adenosine stress (I) vascular permeability in patients treated with VEGFI monotherapy and combined VEGFI with immunotherapy.

**
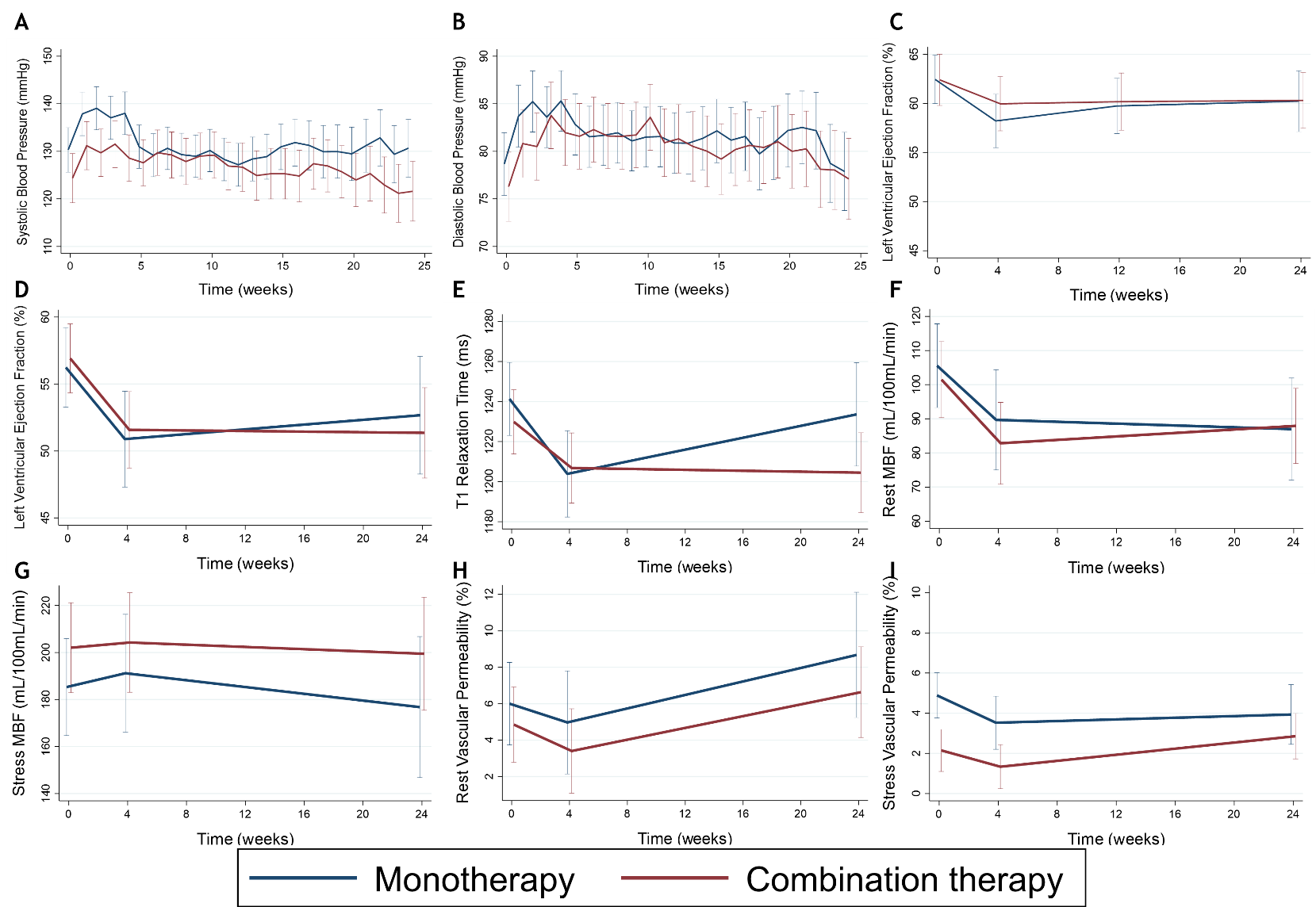
**

**Supplementary Figure 6.** Left ventricular ejection fraction by CMR (A), T1 relaxation times (B) and resting (C) and adenosine stress (D) myocardial in patients who developed VEGFI-associated CTRCD and those who did not.


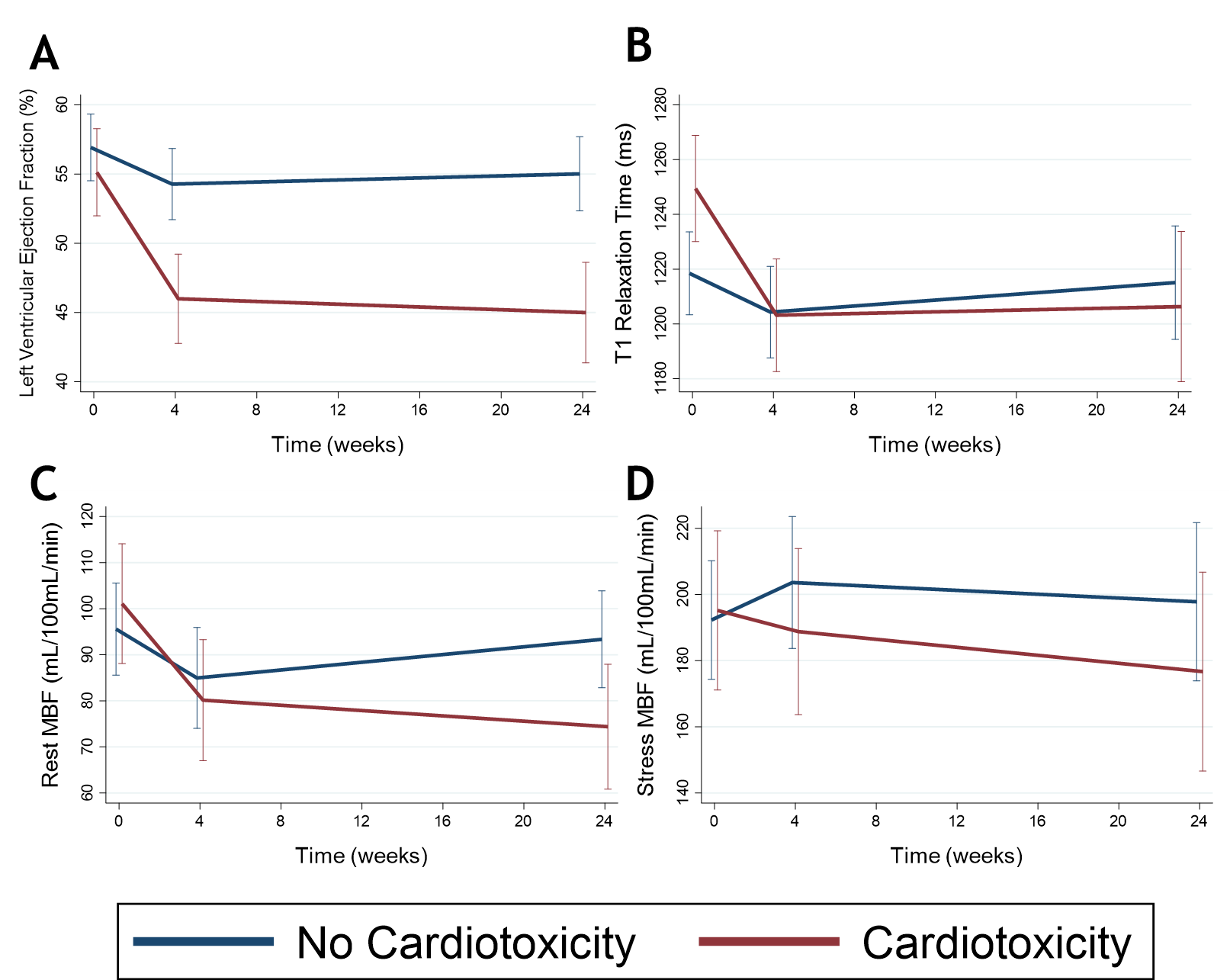


**Table legends**

**Supplementary Table 1.** Outcomes of patients treated with VEGFI therapy. CMR – cardiac magnetic resonance imaging; CTRCD – cancer therapy-related cardiac dysfunction; DBP – diastolic blood pressure; hs-TnT – high sensitivity troponin T; LVEF – left ventricular ejection fraction; LV GLS – left ventricular global longitudinal strain; MBF – myocardial blood flow; NT-proBNP – N-terminal pro-brain natriuretic peptide; SBP – systolic blood pressure.

**Supplementary Table 2.** Baseline characteristics of study and sub-study patients. ACEi – angiotensin-converting enzyme inhibitor; ARB – angiotensin receptor blocker; BMI – body mass index; CABG – coronary artery bypass grafting; CCB – calcium channel blocker; DBP – diastolic blood pressure; HFA-ICOS – Heart Failure Association – International Cardio-Oncology Society; HR – heart rate; hs-TNT – high sensitivity troponin T; LVEF – left ventricular ejection fraction; LVIDd – diastolic left ventricular internal diameter; MRA – mineralocorticoid receptor antagonist; NT-proBNP – N-terminal pro-brain natriuretic peptide; PCI – percutaneous coronary intervention; SBP – systolic blood pressure; VEGFI – vascular endothelial growth factor inhibitor.

**Supplementary Table 1.** Outcomes of patients treated with VEGFI therapy.

| **Outcome** | | **Baseline to 1 week** | **p-value** | **Baseline to 4 weeks** | **p-value** | **Baseline to 12 weeks** | **p-value** | **Baseline to 24 weeks** | **p-value** |
| --- | --- | --- | --- | --- | --- | --- | --- | --- | --- |
| Echo  LVEF (%) | All patients |  |  | -4.2  (-6.2 to -2.3) | <0.001 | -2.3 (-4.4 to -0.2) | 0.034 | -2.0 (-4.2 to 0.1) | 0.067 |
|  |  |  |  |  |  |  |  |  |  |
|  | CTRCD |  |  | -11.3  (-15.9 to -6.6) | <0.001 | -4.7 (-9.7 to 0.3) | 0.066 | -3.0 (-8.4 to 2.4) | 0.267 |
|  |  |  |  |  |  |  |  |  |  |
|  | No CTRCD |  |  | -2.1 (-4.2 to 0.0) | 0.054 | -1.4 (-3.6 to 0.9) | 0.226 | -1.5 (-3.9 to 0.9) | 0.214 |
|  |  |  |  |  |  |  |  |  |  |
| Echo  LVGLS (%) | All patients |  |  | 1.3 (0.3 to 2.2) | 0.007 | 1.0 (-0.1 to 2.1) | 0.073 | 1.8 (0.2 to 3.4) | 0.028 |
|  |  |  |  |  |  |  |  |  |  |
|  | CTRCD |  |  | 3.7 (1.5 to 5.8) | 0.001 | 1.2 (-1.5 to 3.8) | 0.393 | 1.4 (-2.6 to 5.5) | 0.482 |
|  |  |  |  |  |  |  |  |  |  |
|  | No CTRCD |  |  | 0.5 (-0.5 to 1.4) | 0.365 | 0.8 (-0.4 to 2.0) | 0.205 | 1.5 (-0.3 to 3.3) | 0.1 |
|  |  |  |  |  |  |  |  |  |  |
| Home SBP (mmHg) | | 7.2 (4.7 to 9.8) | <0.001 | 6.1 (3.4 to 8.7) | <0.001 | -0.7 (-4.0 to 2.7) | 0.695 | -1.5 (-6.4 to 3.4) | 0.556 |
| Home DBP (mmHg) | | 4.8 (3.1 to 6.5) | <0.001 | 6.2 (4.4 to 8.0) | <0.001 | 3.5 (1.3 to 5.7) | 0.002 | -0.1 (-3.2 to 3.1) | 0.967 |
| Clinic SBP (mmHg) | |  |  | 2.0 (-1.4 to 5.5) | 0.249 | -2.8 (-6.6 to 1.0) | 0.144 | -0.8 (-5.3 to 3.8) | 0.736 |
|  |  |  |  |  |  |  |  |  |  |
| Clinic DBP (mmHg) | |  |  | 3.3 (1.1 to 5.6) | 0.004 | 1.8 (-0.6 to 4.2) | 0.136 | 0.3 (-2.3 to 2.9) | 0.839 |
|  |  |  |  |  |  |  |  |  |  |
| NT-proBNP (pg/mL) | All patients |  |  | 318 (51 to 584) | 0.02 | 151 (-124 to 425) | 0.28 | 79 (-213 to 371) | 0.595 |
|  |  |  |  |  |  |  |  |  |  |
|  | CTRCD |  |  | 1318 (629 to 2007) | <0.001 | 593 (-111 to 1297) | 0.098 | 370 (-374 to 1115) | 0.327 |
|  |  |  |  |  |  |  |  |  |  |
|  | No CTRCD |  |  | 91 (-214 to 395) | 0.558 | 71 (-239 to 381) | 0.652 | 42 (-285 to 370) | 0.799 |
|  |  |  |  |  |  |  |  |  |  |
| hs-TnT (pg/mL) | All patients |  |  | 1.5 (-1.3 to 4.3) | 0.375 | 2.6 (-0.2 to 5.4) | 0.068 | 1.9 (-1.2 to 4.9) | 0.23 |
|  |  |  |  |  |  |  |  |  |  |
|  | CTRCD |  |  | -2.5 (-5.9 to 0.9) | 0.149 | -2.6 (-6.7 to 1.4) | 0.202 | -2.7 (-8.7 to 3.2) | 0.357 |
|  |  |  |  |  |  |  |  |  |  |
|  | No CTRCD |  |  | 1.4 (-0.6 to 3.4) | 0.175 | 1.6 (-0.9 to 4.0) | 0.212 | 3.3 (-0.1 to 6.8) | 0.059 |
|  |  |  |  |  |  |  |  |  |  |
| CMR  LVEF (%) | All patients |  |  | -5.2 (-7.3 to -3.1) | <0.001 |  |  | -4.8 (-7.1 to -2.5) | <0.001 |
|  |  |  |  |  |  |  |  |  |  |
|  | CTRCD |  |  | -6.5 (-10.4 to -2.6) | 0.001 |  |  | -8.2 (-12.4 to -4.0) | <0.001 |
|  |  |  |  |  |  |  |  |  |  |
|  | No CTRCD |  |  | -2.6 (-5.1 to -0.2) | 0.033 |  |  | -1.9 (-4.4 to 0.6) | 0.13 |
|  |  |  |  |  |  |  |  |  |  |
| T1 (ms) | All patients |  |  | -27 (-40 to -14) | <0.001 |  |  | -18 (-33 to -2) | 0.025 |
|  |  |  |  |  |  |  |  |  |  |
|  | CTRCD |  |  | -32 (-58 to -6) | 0.017 |  |  | -40 (-70 to -9) | 0.013 |
|  |  |  |  |  |  |  |  |  |  |
|  | No CTRCD |  |  | -14 (-31 to 2) | 0.089 |  |  | -3 (-22 to 15) | 0.71 |
|  |  |  |  |  |  |  |  |  |  |
| Resting MBF (mL/100mL/min) | All patients |  |  | -14.7 (-24.2 to -5.1) | 0.004 |  |  | -11.4 (-22.0 to -0.9) | 0.034 |
|  |  |  |  |  |  |  |  |  |  |
|  | CTRCD |  |  | -10.6 (-22.8 to -0.6) | 0.047 |  |  | -24.5 (-44.7 to -4.2) | 0.019 |
|  |  |  |  |  |  |  |  |  |  |
|  | No CTRCD |  |  | -10.3 (-29.5 to 8.9) | 0.287 |  |  | -2.2 (-14.6 to 10.2) | 0.719 |
|  |  |  |  |  |  |  |  |  |  |
| Stress MBF (mL/100mL/min) | All patients |  |  | 4.4 (-10.8 to 19.7) | 0.562 |  |  | -3.9 (-21.6 to 13.8) | 0.66 |
|  |  |  |  |  |  |  |  |  |  |
|  | CTRCD |  |  | -17.7 (-49.2 to 13.8) | 0.261 |  |  | -24.0 (-60.0 to 12.0) | 0.184 |
|  |  |  |  |  |  |  |  |  |  |
|  | No CTRCD |  |  | 11.3 (-8.4 to 31.0) | 0.251 |  |  | 5.5 (-17.1 to 28.1) | 0.624 |
|  |  |  |  |  |  |  |  |  |  |

**Supplementary Table 2.** Baseline characteristics of study and sub-study patients.

|  | All patients  n=78 | CMR sub-study  n=46 |
| --- | --- | --- |
| Age, years; mean ± SD | 63 ± 11 | 62 ± 10 |
| Sex, no. (%)  Female | 25 (32) | 9 (20) |
| BMI, Kg/m^2^;  median (IQR) | 27  (25 – 31) | 27  (24 – 30) |
| Cancer primary site,  no. (%)  Renal cell  Hepatocellular  Sarcoma  Thyroid | 56 (72)  13 (17)  5 (6)  4 (5) | 38 (83)  4 (9)  3 (6)  1 (2) |
| Metastases, no. (%) | 67 (86) | 42 (91) |
| Previous cancer treatment, no. (%)  Surgery  Radiotherapy  Chemotherapy | 49 (63)  13 (17)  12 (15) | 31 (67)  8 (17)  10 (22) |
| HR, bpm; mean ± SD | 77 ± 13 | 78 ± 13 |
| Home SBP, mmHg;  mean ± SD | 128 ± 14 | 130 ± 13 |
| Home DBP, mmHg;  mean ± SD | 78 ± 10 | 80 ± 9 |
| LVEF, %; mean ± SD | 63 ± 8 | 63 ± 7 |
| LVIDd, mm; mean ± SD | 46 ± 5 | 46 ± 5 |
| hs-TnT, pg/mL;  median (IQR) | 9.7  (6.1 – 15.4) | 9.9  (6.4 – 15.5) |
| NT-proBNP, pg/mL;  median (IQR) | 132  (57 – 289) | 82  (40 – 202) |
| HFA-ICOS risk assessment, no. (%)  Low  Medium  High  Very high | 19 (24)  24 (31)  17 (22)  18 (23) | 12 (27)  16 (36)  11 (25)  5 (11) |
